# Modelling of aortic aneurysm and aortic dissection through 3D printing

**DOI:** 10.1002/jmrs.212

**Published:** 2017-01-30

**Authors:** Daniel Ho, Andrew Squelch, Zhonghua Sun

**Affiliations:** ^1^ Department of Medical Radiation SciencesCurtin UniversityPerthWestern AustraliaAustralia; ^2^ Department of Exploration GeophysicsWestern Australian School of MinesCurtin UniversityPerthWestern AustraliaAustralia; ^3^ Pawsey Supercomputing CentreKensingtonWestern AustraliaAustralia

**Keywords:** 3D printing, aortic aneurysm, aortic dissection, image processing

## Abstract

**Introduction:**

The aim of this study was to assess if the complex anatomy of aortic aneurysm and aortic dissection can be accurately reproduced from a contrast‐enhanced computed tomography (CT) scan into a three‐dimensional (3D) printed model.

**Methods:**

Contrast‐enhanced cardiac CT scans from two patients were post‐processed and produced as 3D printed thoracic aorta models of aortic aneurysm and aortic dissection. The transverse diameter was measured at five anatomical landmarks for both models, compared across three stages: the original contrast‐enhanced CT images, the stereolithography (STL) format computerised model prepared for 3D printing and the contrast‐enhanced CT of the 3D printed model. For the model with aortic dissection, measurements of the true and false lumen were taken and compared at two points on the descending aorta.

**Results:**

Three‐dimensional printed models were generated with strong and flexible plastic material with successful replication of anatomical details of aortic structures and pathologies. The mean difference in transverse vessel diameter between the contrast‐enhanced CT images before and after 3D printing was 1.0 and 1.2 mm, for the first and second models respectively (standard deviation: 1.0 mm and 0.9 mm). Additionally, for the second model, the mean luminal diameter difference between the 3D printed model and CT images was 0.5 mm.

**Conclusion:**

Encouraging results were achieved with regards to reproducing 3D models depicting aortic aneurysm and aortic dissection. Variances in vessel diameter measurement outside a standard deviation of 1 mm tolerance indicate further work is required into the assessment and accuracy of 3D model reproduction.

## Introduction

Three‐dimensional (3D) printing, also known as rapid prototyping, has seen increasing use in medicine, which has allowed the generation of physical models that can accurately depict complex anatomy in cardiovascular disease.^1^, ^2^ While 3D reconstructions can be generated from computed tomography (CT) or magnetic resonance imaging (MRI), they are limited by an overall lack of realism, require a computer screen for viewing and have an inability to be physically manipulated.^3^ The 3D printed model gives the clinician an opportunity to develop a more intuitive understanding of complex cardiovascular detail and structural abnormalities, in comparison to a computer‐generated 3D reconstruction.

The medical benefits of individualised 3D printed models include: assisting clinical diagnosis, choosing the best operative strategy, predicting any intra‐operative challenges in advance, education and training for junior surgeons, and generating customisable prostheses and implants to suit the individual patient.^1^, ^4^, ^5^, ^6^, ^7^ Some recent studies have shown the applications of 3D printing in cardiovascular disease, such as coronary artery disease, aortic and pulmonary venous valve disease.^8^, ^9^, ^10^, ^11^, ^12^ 3D printing technology also allows for the production of individualised cardiac stents to reduce the rate of in‐stent re‐stenosis.^1^

However, no report is available in the literature with regard to the use of 3D printing in accurately producing physical models of aortic dissection and aortic aneurysm involving the aortic arch. The rationale for choosing these two pathologies in this study is that both aortic diseases represent common cardiovascular diseases which are associated with high morbidity and mortality. Due to complex anatomy in the aortic region, in particular, the area of aortic arch and thoracic aorta with a number of important arterial branches arising from the aortic arch, it is still difficult to appreciate the real 3D relationship between aortic disease and these arterial branches with conventional CT images. 3D printing holds promise in demonstrating the 3D relationship between aortic dissection and aortic aneurysm and arterial branches, thus, providing guidance for clinical management of these life‐threatening cardiovascular disease, in particular pre‐operative training and simulation of endovascular stent grafting procedures. Treatment of type B dissection is controversial because it can be managed by surgery, endovascular repair, or medical treatment depending on clinical presentation. If patients are asymptomatic with no complications such as rapid progression of the dissecting aneurysm or malperfusion, best medical treatment is suggested, whereas for patients presenting with symptoms or developing complications such as rupture, malperfusion syndrome or aortic insufficiency, surgical intervention is recommended. However, optimal timing and treatment modality remains a challenging issue for clinicians to make an important decision for this group of patients, and this is the subject of current debate. 3D printed patient‐specific aortic models will enable direct visualisation and assessment of anatomical features of aortic dissection including the size and shape of true and false lumens.

Thus, in this study we present our preliminary experiences of developing 3D printed models of aortic dissection and aortic aneurysm involving the aortic arch, generated from the contrast‐enhanced CT scans of two patients. The aim of this study was to assess whether the complex anatomy of these diseases can be physically reproduced in an accurate manner. This could allow clinicians to confidently conduct pre‐operative planning and simulate procedures on the model, such as aneurysm resection or endovascular stent graft repair.^13^, ^14^

## Materials and Methods

### Selection of sample cases for image post‐processing

The CT examinations of two patients, one with an aortic aneurysm (Model 1) and the other a Stanford type B aortic dissection (Model 2) involving the aortic arch, were selected. The contrast‐enhanced cardiac CT images, saved in the Digital Imaging and Communications in Medicine (DICOM) format, were imported into *Analyze 12.0* (AnalyzeDirect Inc., Lexana, KS, USA), for image post‐processing and segmentation. The CT data were visualised using 3D surface rendering with the minimum threshold value set to 200 HU. This threshold technique facilitated the removal of low attenuation anatomy (such as soft tissue) from the data, allowing clear visualisation of high attenuation anatomy (such as the contrast‐filled thoracic aorta and bone). Since only de‐identified CT images were used for generation of 3D printed models, ethical approval/patient informed consent was waived due to the retrospective nature of data collection.

**Figure 1:**
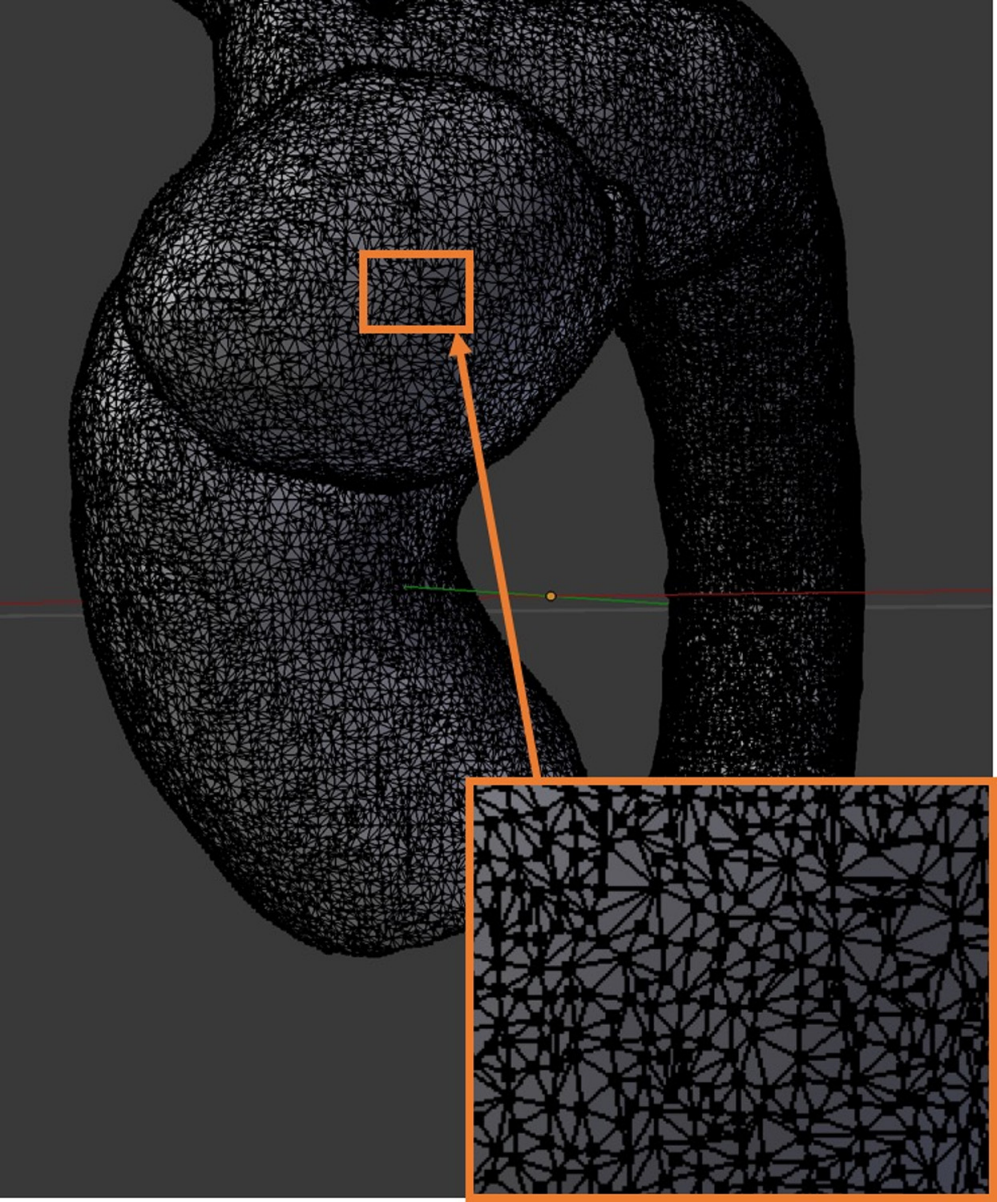
Demonstration of the triangular mesh in Model 1.

Following 3D surface rendering, automatic and manual segmentation methods were used so that only the thoracic aorta and the base of the brachiocephalic, left common carotid, and left subclavian arteries as well as aortic aneurysm were visible. The data sets were exported in Standard Tessellation Language (STL) format into a 3D triangular mesh, that is the surface contours of the model were approximated with a connected series of triangular faces (Fig. 1).^15^

**Figure 2:**
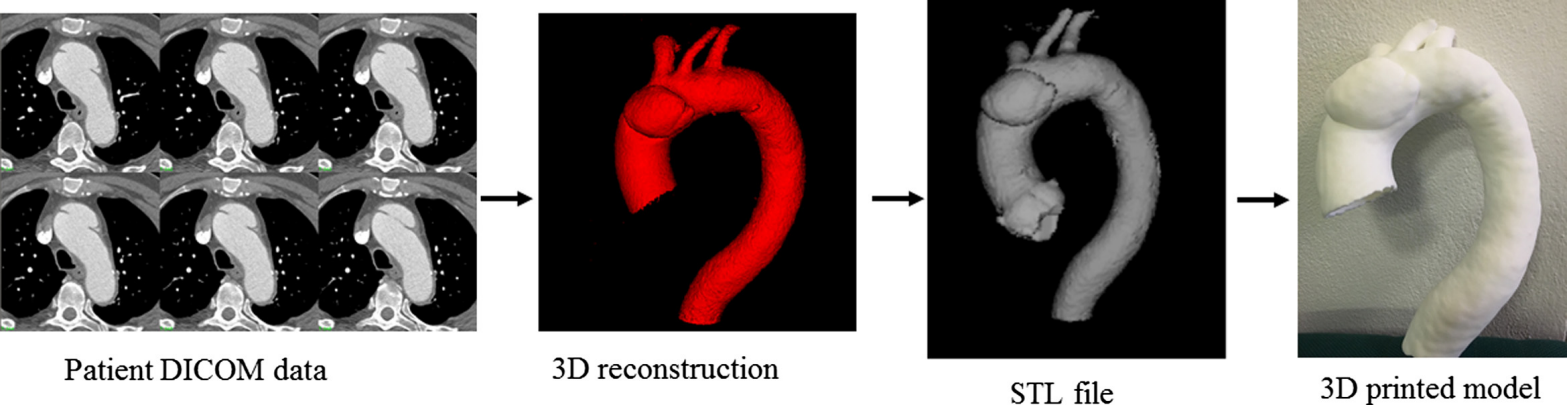
Flow diagram showing the progress from original DICOM CT data to a 3D depiction of the aorta, generation of STL file for 3D printing.

The STL file exported from *Analyze* was then imported into *Blender,* a free and open‐source Computer‐Aided Design (CAD) software package, to further refine the triangular mesh. *Blender* is developed by the Blender Foundation, a Dutch public‐benefit corporation establishing and supporting the *Blender* software, in conjunction with its users.^16^ Using *Blender*, the triangular faces covering the ends of the ascending aorta, descending aorta, brachiocephalic artery, left common carotid and left subclavian arteries of each aorta model were removed. This allowed the inside of each model to be visible. A 2.5 mm maximum, non‐uniform external wall thickness was applied to the models, meaning that external wall thickness at any point around the model was equal to or less than 2.5 mm. The 2.5 mm figure approximated the thickness of the aortic wall based on the patient CT data. While achieving a 2.5 mm uniform wall thickness would have been an ideal outcome, significant deformities occurred in the model when this was attempted in *Blender* and it was impractical to 3D print the resulting model. A smoothing filter was also applied to the models to give a smoother appearance and feel when physically printed. Figure 2 is a flow diagram showing the stages from image post‐processing and segmentation of CT data to generation of STL and 3D printing.

The 3D volume rendering of the aorta of the patient with the Stanford B dissection was further edited slice‐by‐slice from an axial view to delineate the intimal flap separating the true and false lumens. The intimal flap was created in two small separate regions, where a clear separation between the true and false lumen was seen on the surface render.

### 3D printing

The *Blender*‐edited STL file for each model was uploaded onto *Shapeways,* an online 3D printing service enabling people to make, buy and sell 3D printed products, with production facilities located in New York, USA, and Eindhoven, the Netherlands.^17^ When a 2.5 mm maximum, non‐uniform external wall thickness was applied in *Blender,* most walls in both models were between 0.7 mm (Shapeways’ minimum printing requirement) and 2.5 mm thick. However, small areas of the model contained walls less than 0.7 mm thick, so the Shapeways’ ‘Fix Thin Walls’ function was applied to ensure these walls met the minimum wall thickness requirements. After this step, both models were submitted for printing in ‘Strong and Flexible Plastic’.^18^ Shapeways uses Selective Laser Sintering technology to print in this material, which uses a laser to fuse together nylon powder, layer‐by‐layer.^18^ As reported earlier by others, Selective Laser Sintering allows for large part sizes and has a good degree of strength, but it produces a rough, powdery surface which requires additional sealing and sanding to improve the finish.^19^, ^20^

**Figure 3:**
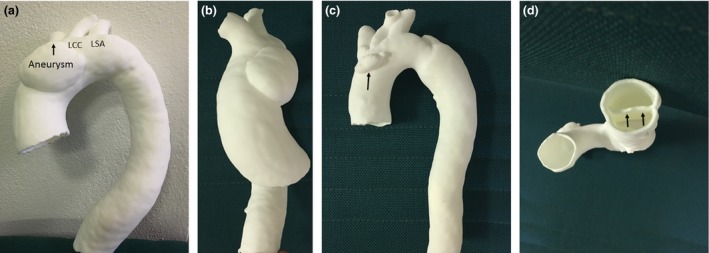
3D printed models generated from cardiac CT images. (A) Model 1 showing aortic aneurysm relative to the three arterial branches arising from the aortic arch, namely LSA‐left subclavian artery, LCC‐left common carotid artery and innominate artery (arrow). (B) lateral view of Model 1 showing the aneurysm. (C) anterior view of Model 2 with artefact (arrow) in the aortic arch due to image post‐processing. (D) caudocranial view of aortic dissection showing intimal flap (arrows).

**Figure 4:**
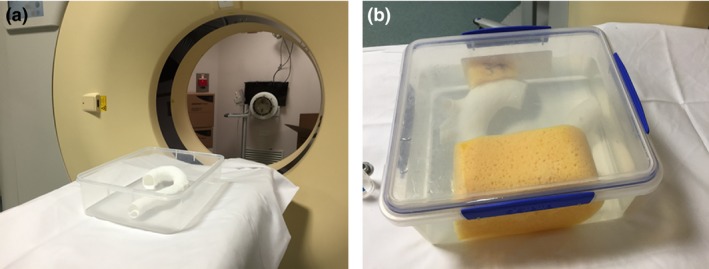
Preparing one 3D‐printed aorta model for a post‐contrast scan (A). The model was immersed in approximately 3 L of fluid (80 mL Ultravist, approximately 2920 mL water) with sponges placed to immobilise the model during scanning (B).

When the models were physically produced, non‐contrast and contrast‐enhanced CT scans were performed for each 3D printed model (Fig. 3). This was conducted on a 256 CT scanner (iCT; Philips Medical Systems, Best, the Netherlands) at 100 kV and 100 mAs, using a thoracic aorta angiographic protocol, resulting in a voxel size of 0.68 × 0.68 × 0.68 mm^3^. A similar voxel size of 0.65 × 0.65 × 0.65 mm^3^ was acquired with the original CT angiographic protocol performed on a 128‐slice CT scanner (Somatom Definition Flash, Siemens Healthcare, Forchheim, Germany) at 100 kV and 330 mAs. For the contrast‐enhanced scans, each model was immersed in a plastic container filled with approximately 3L of fluid. This comprised of 80 mL Ultravist (Bayer Australia Ltd, Pymble, NSW, Australia), with water making up the remaining volume (Fig. 4).

### Measurements of anatomic accuracy

The internal transverse diameter, that is, the luminal diameter, was measured from left to right (LR) and from anterior to posterior (AP) at five anatomical landmarks for both models. These anatomical landmarks were the ascending aorta, descending aorta, and the base of the brachiocephalic, left common carotid and left subclavian arteries. Measurements of internal transverse diameter were taken at three stages of the model generation process, with all measurements being conducted by the same observer.

The mean difference in vessel diameter was calculated by combining the means of the left‐right and anterior‐posterior differences, at the five anatomical landmarks (Table 1). This calculation was carried out for the patient's CT scan and the CT scan of the 3D printed model. The differences presented in Tables 1 and 2 are absolute values. For this study, the acceptable vessel diameter difference between the patient's CT and the CT of the 3D printed model was considered as 1 mm or less; using a tolerance as small as practicable in this study was important to determine whether models of high accuracy can be produced.

**Table 1:** Difference in vessel dimensions for the three aorta models when comparing the patient CT scan, STL file and the CT scan of the 3D model. The differences presented are absolute values

Landmark	Difference in diameter of vessel (mm)											
	STL file compared to patient CT (Comparing A and B)				3D print CT compared to STL file (Comparing B and C)				3D print CT compared to patient CT (Comparing A and C)			
	Model 1		Model 2		Model 1		Model 2		Model 1		Model 2	
	LR	AP	LR	AP	LR	AP	LR	AP	LR	AP	LR	AP
Descending aorta	0.2	0.3	2.1	1.1	0.5	1.7	0.8	0.2	0.3	1.4	1.3	1.3
Ascending aorta	2.5	0.2	0.0	2.8	2.6	0.8	0.7	0.6	0.1	0.6	0.7	0.6
Brachiocephalic artery	2.5	2.1	0.1	0.4	0.3	0.4	0.3	0.1	2.2	1.7	0.4	0.3
Left common carotid artery	0.6	1.2	0.4	1.7	0.8	1.8	0.8	0.5	0.2	3.0	1.2	1.2
Left subclavian artery	0.7	0.4	0.3	2.3	1.2	0.5	1.5	0.9	0.5	0.1	1.8	3.2
Mean difference	1.1		1.1		1.1		0.6		1.0		1.2	
Standard deviation	1.0		1.0		0.8		0.4		1.0		0.9	

STL, stereolithography; CT, computed tomography; LR, left to right; AP, anterior to posterior.

**Table 2:** Difference in true and false lumen diameter for Model 2 when comparing the patient CT scan, computerised model and the CT scan of the 3D model. The differences presented are absolute values

Landmark	Difference in luminal diameter (mm)		
	Model 2: Aortic dissection		
	STL file compared to patient CT (Comparing A and B)	3D print CT compared to STL file (Comparing B and C)	3D print CT compared to patient CT (Comparing A and C)
Descending aorta, point 1^a^			
True lumen	0.1	1.1	1.0
False lumen	0.7	0.4	0.3
Descending aorta, point 2^a^			
True lumen	0.1	0.6	0.5
False lumen	0.1	0.1	0.2
Mean difference	0.3	0.6	0.5

STL, stereolithography; CT, computed tomography.

a Point 1 on the descending aorta was defined as the axial slice going through the most inferior point of the model's ascending aorta. Point 2 on the descending aorta was defined as the most inferior point of the model's descending aorta.

**Figure 5:**
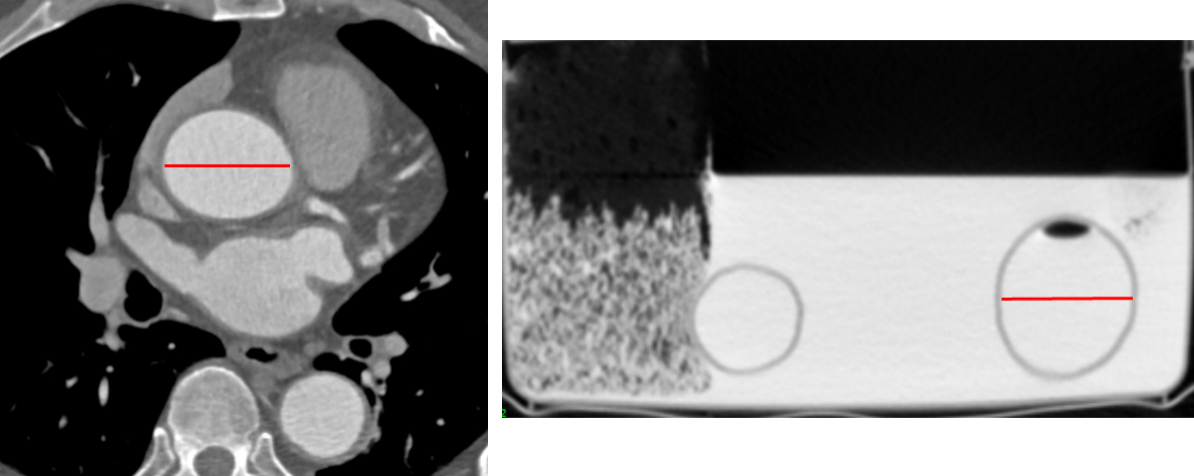
(Left) Measuring the transverse diameter of the ascending aorta in the patient's CT scan using the line tool in Analyze software. (Right) Measuring the transverse diameter of the descending aorta in the contrast‐enhanced CT scan of the 3D printed model using the line tool in Analyze software.

Firstly, the contrast‐enhanced CT scan of the patient was measured using *Analyze*'s measurement function. Secondly, the computerised, STL‐format model was measured using the measurement tool in *Blender*. Finally, the contrast‐enhanced CT scan of the 3D printed model was measured in *Analyze*. Across the three stages, the measurements were compared to determine whether the dimensions of the patient's aortic lumen as per the CT scan of the patient, can be accurately reproduced in the computerised and 3D printed model (Fig. 5).

In addition, for Model 2, which featured an aortic dissection, the dimensions of the true lumen and false lumen were measured at two points, over the same three stages of the model generation process. The measurements were compared across the three stages to assess whether the patient's intimal flap can be accurately reproduced in the computerised and 3D printed model.

## Results

### Replication of dimensions of aortic lumen

The mean difference in vessel diameter was 1.0 and 1.2 mm for Model 1 and 2, respectively, when comparing the contrast‐enhanced CT of the 3D printed model to the patient (Table 1). The standard deviation was recorded at 1.0 mm and 0.9 mm for Model 1 and 2 respectively. Differences in vessel diameters ranged from nil to 3.2 mm (Table 1).

### Replication of intimal flap

When comparing the contrast‐enhanced CT of the 3D printed model to the CT of the patient at two points on the descending aorta, measuring the true and false lumens, the mean difference in luminal diameter was only 0.5 mm (Table 2). Similarly, good accuracy was also obtained when comparing the computerised model to the pre‐3D printing contrast‐enhanced CT images (mean luminal difference: 0.3 mm), and when comparing the contrast‐enhanced CT of the 3D printed model to the computerised model (mean luminal difference: 0.6 mm) (Table 2). The separation could not be visualised throughout the entire length of the descending thoracic aorta due to a very thin fibrous structure of the intimal flap, so a continuous intimal flap could not be reproduced in the 3D printed model as per the original patient CT scan.

## Discussion

From these preliminary experiences, this study shows that 3D printing can be used to accurately replicate anatomical details of aortic aneurysm and dissection, including the intimal flap, compared to the pre‐3D printing CT images. Encouraging results were yielded when reproducing the intimal flap, with the mean difference in luminal diameter within 1 mm of error. Previous studies have highlighted that cardiac 3D printed models reduce the risk of perioperative complications because potential challenges can be anticipated through simulating procedures on the model, such as transcatheter aortic valve replacement.^9^, ^11^ 3D printed models also allow for increased procedural efficiency, as well as improved anatomical understanding and intraoperative orientation.^8^, ^12^

The 3D printed models of aortic dissection and aortic aneurysm involving the aortic arch developed in this study has potential value in simulating surgical procedures like endovascular stent grafting, which can facilitate more precise procedural planning. These models would also be beneficial for teaching or educational purposes, such as giving junior or inexperienced surgeons a better appreciation of complex aortic anatomy. Furthermore, these models could give the affected patient and their families a more tangible understanding of their condition.

The most important comparison in this study was the vessel diameter measurements of the patient's CT versus the contrast‐enhanced CT of the 3D printed model. Recording measurements of the computerised model in *Blender* was useful in showing the progress of changes in vessel diameter across the model development process. A high degree of anatomical accuracy in the 3D printed models is important; without a high degree of accuracy, clinicians may not be able to confidently conduct accurate pre‐procedure planning on the model, such as aneurysm resection. As aforementioned, a 1 mm or less difference between the patient's CT and the CT of the 3D printed model was considered acceptable. Using a smaller tolerance less than 1 mm would have not been appropriate due to the limitations in detail of the 512 × 512 pixel matrix of each CT slice.

While numerous vessel diameter differences were recorded at 1 mm or less, it must be acknowledged that there were large standard deviations, with differences up to 3.2 mm recorded. The variances in vessel diameter difference observed across the three stages of the model generation process – contrast‐enhanced CT of patient, STL‐format computerised model, and the contrast‐enhanced CT of the 3D printed model – likely comes down to three main reasons.

First, it is probable that the models were measured at slightly different points from one stage to the next, although efforts were made to ensure that this was minimised. This was because a mixture of sources (CT scan of patient, computerised model, CT scan of physical model) were used for measurement, which could not be perfectly compared between each other. In future investigations, attempts should be made to align the CT volumes of the patient and the 3D printed model in *Analyze,* in order to draw more meaningful comparisons between the two sources.

Second, the models were not measured using the same software in all three stages. While *Analyze* was used to measure the vessel diameter of the contrast‐enhanced CT scans of the physical model and patient, *Blender* was used to measure the dimensions of the computerised model. Ideally, all measurements should have been conducted in *Analyze* (or in the same programme), however, *Analyze* could not import the STL file format of the computerised model.

Lastly, when importing the STL file (exported from *Analyze*) into *Blender,* the scale was not preserved. As Valentan et al. explains, the STL file format does not contain any information pertaining to scale.^21^ This presented challenges in ensuring the computerised model was an accurate representation of the patient's contrast‐enhanced CT. To counteract this issue, the computerised model and the patient's CT images were measured at the five aforementioned anatomical landmarks, with an average scale factor being calculated from the measurement differences between the two sources. The scale factor was then applied to the computerised model in *Blender*.

Although comparing the patient's CT scan, the 3D computerised model and the CT scan of the 3D printed model at five set points was an indicator of whether anatomical accuracy was preserved, it does not necessarily reflect the accuracy of the 3D printed model in its entirety. The measurements at these five set points were taken only once for each source; repeated measurements at the same points should be taken in future investigations. Furthermore, the large variance in vessel wall thickness (0.7–2.5 mm) would be a limitation if the vessel walls, rather than the aortic lumen, were of particular importance to the clinician in pre‐operative planning.

In terms of replication of the intimal flap in Model 2, the results obtained suggest that the slice‐by‐slice editing method used can faithfully reproduce the patient's intimal flap. However, for this model, the full length of the intimal flap could not be reproduced in the 3D printed model, as per the patient's CT scan. This is because the intimal flap could only be created for regions where the 3D volume rendering view identified a clear separation between the true and false lumen. The 512 × 512 pixel matrix of each CT image slice may have provided inadequate detail to effectively segment out a continuous intimal flap; obtaining higher resolution CT slices may have allowed for improved, more continuous segmentation. Additionally, the editing process was time‐consuming, taking approximately 6 h for this model. While Model 2 in its current iteration would not be suitable for clinicians to confidently simulate procedures, the use of higher resolution CT imaging protocols in future studies would be a step forward in producing a more anatomically representative model of aortic dissection.^22^, ^23^

Although encouraging results were demonstrated in this study, it cannot be confidently determined from these findings alone whether 3D models of aortic aneurysm and dissection can be accurately created on a wider clinical scale for simulation of surgical procedures, due to the limited sample size of this study. Further investigations need to be conducted in creating complex internal aortic structures, such as the intimal flap characteristic of aortic dissection, in a way that is both accurate and not time‐consuming.

## Conclusion

This study demonstrated that models depicting aortic aneurysm and aortic dissection can be physically reproduced from a patient's contrast‐enhanced CT scan into a 3D printed physical model, which could have useful surgical and educational applications. Encouraging results were achieved with regards to reproducing the intimal flap in aortic dissection. However, variances in vessel diameter measurement outside a standard deviation of 1 mm tolerance indicates that further work is required to assess and increase confidence in 3D model reproduction. Further investigations need to be conducted in accurately replicating complex internal aortic detail.

## Conflict of Interest

The authors declare no conflict of interest.
